# Parental concerns during COVID-19-related school closures: children’s behaviors and media usage

**DOI:** 10.1192/j.eurpsy.2022.382

**Published:** 2022-09-01

**Authors:** S.J. Kim, S. Cho, D. Kim

**Affiliations:** 1 Ajou University School of Medicine, Psychiatry, Suwon, Republic of Korea; 2 Myungi University, Health Economics, Seoul, Republic of Korea

**Keywords:** Covid-19, school closure, Parental concern, Media usage

## Abstract

**Introduction:**

Coronavirus Disease 2019 has prompted widespread school closures and physical distancing measures. Concerns regarding COVID-19 school closures often increase stress levels in parents.

**Objectives:**

This study examined whether higher levels of parental concerns were associated with children’s problematic behaviors and other factors during COVID-19-related primary school closures.

**Methods:**

Participants were 217 parents who responded to a web-based questionnaire covering parental concerns, subjective stress, and depression; children’s sleep patterns, behavioral problems, and changes in activity level after COVID-19; previously received mental health services; and media usage during the online-only class period from community center in Suwon city.

**Results:**

The number of parental concerns was associated with children’s behavioral problem index (BPI) score (Pearson correlation 0.211, p < 0.01), sleep problems (0.183, p < 0.01), increased smartphone usage (0.166, p < 0.05), increased TV usage (0.187, p < 0.01), parents’ subjective stress levels (0.168, p < 0.05), and parental depression (0.200, p < 0.01). In families with children who previously received mental health services, the children reportedly suffered from more sleep and behavioral problems but not increased media usage, and parents noted more stress and depression. Parental concerns are related to family factors such as change of caregiver, no available caregiver, decreased household income, and recent adverse life events. Economically vulnerable people also reported higher rates of reported caregiver burden during COVID-19-related primary school closures, thus will require the potential heightened mental health needs.

**Conclusions:**

Ongoing monitoring of mental health at risky group and multiple support systems should be considered for parents having difficulty in caring their children.

**Disclosure:**

No significant relationships.

